# The Integrated Amendment of Sodic-Saline Soils Using Biochar and Plant Growth-Promoting Rhizobacteria Enhances Maize (*Zea mays* L.) Resilience to Water Salinity

**DOI:** 10.3390/plants10091960

**Published:** 2021-09-20

**Authors:** Yasser Nehela, Yasser S. A. Mazrou, Tarek Alshaal, Asmaa M. S. Rady, Ahmed M. A. El-Sherif, Alaa El-Dein Omara, Ahmed M. Abd El-Monem, Emad M. Hafez

**Affiliations:** 1 Department of Agricultural Botany, Faculty of Agriculture, Tanta University, Tanta 31527, Egypt; 2 Citrus Research and Education Center, Department of Plant Pathology, University of Florida, 700 Experiment Station Rd., Lake Alfred, FL 33850, USA; 3 Business Administration Department, Community College, King Khalid University, Guraiger, Abha 62529, Saudi Arabia; ymazrou@kku.edu.sa or; 4 Department of Agriculture Economic, Faculty of Agriculture, Tanta University, Tanta 31527, Egypt; 5 Agricultural Botany, Plant Physiology and Biotechnology Department, University of Debrecen, AGTC, 4032 Debrecen, Hungary; alshaaltarek@gmail.com or; 6 Soil and Water Department, Faculty of Agriculture, University of Kafrelsheikh, Kafr El-Sheikh 33516, Egypt; 7 Crop Science Department, Faculty of Agriculture (EL-Shatby), Alexandria University, Alexandria 21545, Egypt; asmaa.mohamed@alexu.edu.eg; 8 Department of Agronomy, Faculty of Agriculture, Fayoum University, Fayoum 63514, Egypt; ama16@fayoum.edu.eg; 9 Department of Microbiology, Soils, Water and Environment Research Institute, Agricultural Research Center, Giza 12112, Egypt; ala.emara@yahoo.com; 10 Department of Agronomy, Faculty of Agriculture, New Valley University, New Valley, Elkharrga 72511, Egypt; abdelmonem7@gmail.com; 11 Department of Agronomy, Faculty of Agriculture, Kafrelsheikh University, Kafr El-Sheikh 33516, Egypt; emadhafez2012@agr.kfs.edu.eg or

**Keywords:** salinity, sodicity, K^+^/Na^+^, soil enzymes, ESP, water productivity, maize, saline water

## Abstract

The utilization of low-quality water or slightly saline water in sodic-saline soil is a major global conundrum that severely impacts agricultural productivity and sustainability, particularly in arid and semiarid regions with limited freshwater resources. Herein, we proposed an integrated amendment strategy for sodic-saline soil using biochar and/or plant growth-promoting rhizobacteria (PGPR; *Azotobacter chroococcum* SARS 10 and *Pseudomonas koreensis* MG209738) to alleviate the adverse impacts of saline water on the growth, physiology, and productivity of maize (*Zea mays* L.), as well as the soil properties and nutrient uptake during two successive seasons (2018 and 2019). Our field experiments revealed that the combined application of PGPR and biochar (PGPR + biochar) significantly improved the soil ecosystem and physicochemical properties and K^+^, Ca_2_^+^, and Mg_2_^+^ contents but reduced the soil exchangeable sodium percentage and Na^+^ content. Likewise, it significantly increased the activity of soil urease (158.14 ± 2.37 and 165.51 ± 3.05 mg NH_4_^+^ g^−1^ dry soil d^−1^) and dehydrogenase (117.89 ± 1.86 and 121.44 ± 1.00 mg TPF g^−1^ dry soil d^−1^) in 2018 and 2019, respectively, upon irrigation with saline water compared with non-treated control. PGPR + biochar supplementation mitigated the hazardous impacts of saline water on maize plants grown in sodic-saline soil better than biochar or PGPR individually (PGPR + biochar > biochar > PGPR). The highest values of leaf area index, total chlorophyll, carotenoids, total soluble sugar (TSS), relative water content, K^+^ and K^+^/Na^+^ of maize plants corresponded to PGPR + biochar treatment. These findings could be guidelines for cultivating not only maize but other cereal crops particularly in salt-affected soil and sodic-saline soil.

## 1. Introduction

Field crops are constantly exposed to several abiotic stresses, including water scarcity, soil salinity, and irrigation with poor-quality water; all of which can restrict crop productivity by more than 50% and, eventually, threaten universal food security [1]. More than 800 Mha of the world’s lands are saline soil, either by salinity (397 Mha) or sodicity (434 Mha) [2]. The influences of salinity on yield constitute a further menace in arid and semiarid zones owing to insufficient rainfall, high temperature, low water quality, and poor soil management practices [3,4].

The situation is worse in sodic-saline soil where plants suffer from the limited availability of nutrients, water uptake shortage caused by high osmotic pressure, and ion toxicity due to elevated Na^+^ and Cl^−^ ions [5,6]. Another threat to plant performance in sodic-saline soil is soil dispersion and the swelling of clay platelets and aggregates caused by high Na^+^ content [7]. Plant roots suffer from a severe shortage in soil aeration because the dispersed clay particles plug soil pores causing poor ventilation [8,9].

Soil salinity/sodicity and water scarcity are major global conundrums that severely impacts agricultural productivity and sustainability, particularly in arid and semiarid regions [10] with limited freshwater resources. The utilization of low-quality water or slightly saline water in these soils is a challenge to obtain adequate yield. Unfortunately, recent statistics of global soil salinity are poorly and inadequately reported. For instance, based on different data sources, salinity and sodicity have been reported to affect more than 10% of the total arable land [11].

However, we believe that this percentage is not sufficiently accurate and is an outdated estimate [12] since the same report suggested that one billion hectares are covered with saline and/or sodic soils and that between 25% and 30% of irrigated lands are salt-affected [11]. About 25 years ago, saline soils were reported to occupied more than 20% of the total irrigated area worldwide [13]. Thenceforth, the extent of saline soils has dramatically increased to potentially affect more than half of the irrigated lands in some countries [14]. In Egypt, more than one-third of the total cultivated area in the Nile Delta region, which represents approximately 64% of the total agricultural lands, is classified as salt-affected soil [9].

It has been suggested that biochar application is a promising soil amendment approach to mitigate soil contamination via immobilizing heavy metals [15], improving the overall soil quality [16,17], enhancing water-fertilizer productivity [16], and decreasing soil salinity [18] in arid and semi-arid regions. Biochar can be produced from different sources (well-reviewed by Guo et al. [15]). Common biochar feedstocks extend to forest debris, crop residues, food processing waste, and manures [15,19].

Recent reports have demonstrated the potentiality of biochar to augment soil health and plant productivity through improving soil water retention and the phytoavailability of nutrients [20]. Biochar is a carbon-rich product formed by pyrolysis of cellulose-containing biomass [21]. The cation exchange capacity of biochar depends on the pyrolysis treatment [22]. Biochar is characterized by a high ash content, pH, and specific surface area [1].

Likewise, plant growth-promoting rhizobacteria (PGPR) can alleviate the hazardous impacts of almost all biotic and abiotic stresses, particularly soil problems, such as salinity, and strengthen the resistance of plants through several mechanisms, including enhancing the solubilization of many minerals, biosynthesis of phytohormones, take-up of nutrients and water, and scavenging of oxidants [23]. PGPR-produced phytohormones play a key role in promoting the plant growth and the suppression of biotic and abiotic stress [24]. Most of the PGPR are reported to produce one or more compounds of auxins, particularly indole acetic acid (IAA) [25,26], cytokinins [27], gibberellins [28,29], abscisic acid (ABA) [30], and ethylene [31].

PGPR-derived phytohormones within the vicinity of root promote elongation of primary roots, as well as proliferation of lateral and adventitious roots [24]. Moreover, this increases the root surface area, which enhances the uptake capacity of roots from a large volume of soil and improve the absorbance of water, minerals, and nutrition [24]. Furthermore, PGPR-derived phytohormones enhance the plant survival via strengthening its anchorage capacity [24]. Finally, PGPR-produced phytohormones might enhance the tolerance of plants to adverse abiotic stress. For example, the application of ABA-producing PGPR induced endogenous ABA levels that reduced the harmful effects of drought, salinity, and temperature on treated plants [32].

Maize (*Zea mays* L.) is one of the most important cereals worldwide. It is known as a moderately sensitive crop to salinity up to 1.7 dS m^−1^ of electrical conductivity (ECe) of soil saturated paste. Its yield is reduced by 12% for each increase of 1 dS^−1^ m^−1^ of ECe [33]. Maize is an imperative plant in the temperate climatic zone along with the semi-arid zone owing to the massive need for food and livestock feed. In Mediterranean zones, maize production relies greatly on irrigation. In such areas, where freshwater resources are limited, irrigation with low-quality water, such as saline water, is common. Therefore, it could be very imperative to improve irrigation management [34].

To our knowledge, there are few studies that have been conducted on the integrated effect of biochar and PGPR on the growth and physiology of maize under saline water irrigation in sodic-saline soil. In light of these considerations, a field experiment was performed with the following objectives: (a) to determine the integrated effect of biochar and PGPR in mitigating salinity resulting from saline water irrigation and sodic-saline soil to improve the growth, crop physiological processes, and yield of maize and (b) to assess the improvement in soil physicochemical properties due to the application of biochar and PGPR added singly or in combination.

## 2. Results

### 2.1. Biochar and PGBP Application Improved the Soil Ecosystem

#### 2.1.1. Soil Physicochemical Properties

**Figure 1:**
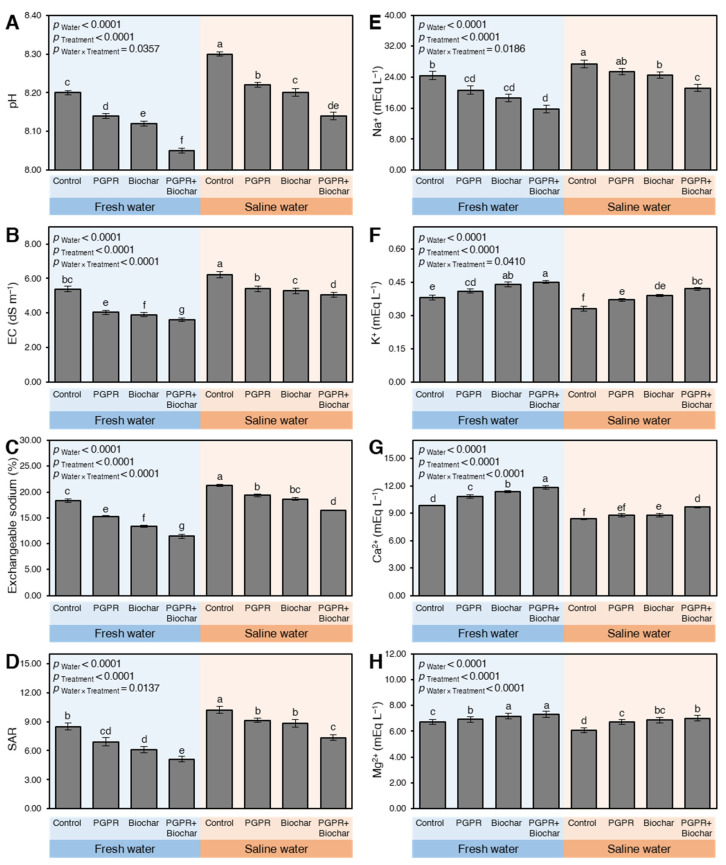
The soil chemical properties at harvest time of maize plants growing in sodic-saline soil and irrigated with fresh and saline water after the application of biochar and PGPR during the 2019 season. Data presented are the means ± standard deviation (mean ± SD) of three biological replicates. Presented pairwise differences connecting letters (significance letters) were generated based on the *p*-value of the interaction between water type (as the main plots) and treatments (as subplots) that were mentioned as (*p*_Water × Treatment_). Means followed by different letters indicate statistically significant differences among treatments according to Tukey’s honestly significant difference (HSD) test (*p* ≤ 0.05), whereas means followed by the same letters indicate no statistically significant differences among them. EC: Electrical conductivity; SAR: Sodium adsorption ratio. mEq L^−1^: milliequivalents per liter.

Although the utilization of saline water to irrigate maize plants in sodic-saline soil significantly increased the soil pH compared to freshwater (*p*_Water_ < 0.0001) in both seasons 2018 (Figure S1A) and 2019 (Figure 1A), the application of PGPR, biochar, or their combination significantly enhanced the soil acidity through declining the soil pH (*p*_Treatment_ < 0.0001). The PGPR + biochar treatment had the lowest pH in both growth seasons 2018 (*p*_Water × Treatment_ = 0.0357) and 2019 (*p*_Water × Treatment_ = 0.0353) (Figure S1A and Figure 1A, respectively) when maize plants were irrigated with freshwater or saline water.

Likewise, irrigating maize plants with saline water considerably increased the EC compared to freshwater (*p*_Water_ < 0.0001) in 2018 (Figure S1B) and 2019 (Figure 1B). Nevertheless, the utilization of PGPR and biochar singularly or in combination significantly reduced the EC regardless of the type of irrigation water (*p*_Treatment_ < 0.0001). PGPR + biochar-treated soil had the lowest EC under both types of irrigation water in both seasons 2018 and 2019 (*p*_Water × Treatment_ < 0.0001 in both seasons). Interestingly, EC values after treating soils that received saline water with PGPR + biochar were comparable and even significantly lower than those of control plants irrigated with freshwater during 2018 and 2019 (Figure S1B and Figure 1B, respectively)

Like pH and EC, both soil ESP (Figure S1C and Figure 1C in 2018 and 2019, respectively) and SAR (Figure S1D and Figure 1D in 2018 and 2019, respectively) exhibited a similar response. Although the utilization of saline water to irrigate maize plants significantly increased the soil ESP and SAR compared to freshwater (*p*_Water_ < 0.0001), PGPR and/or biochar application significantly reduced both ESP and SAR when fresh or saline water was used. Saline soil that received PGPR + biochar and irrigated with freshwater had the lowest ESP (*p*_Water_ _× Treatment_ < 0.0001 in both seasons) and SAR (*p*_Water_ _× Treatment_ = 0.0137 and 0.0129 in 2018 and 2019, respectively) compared with all other treatments. It is worth mentioning that there are no significant differences between the singular treatment of PGPR and biochar in terms of EPS in 2019 (Figure 1C) and SAR in 2018 (Figure S1D).

Similar results were noticed in the concentrations of soil Na^+^ content. Irrigation with saline water significantly increased the soil Na^+^ compared with freshwater (*p*_Water_ < 0.0001). However, the application of PGPR and/ or biochar significantly reduced the soil Na^+^ content (*p*_Treatment_ < 0.0001) with a greater effect of PGPR + biochar treatment in both 2018 (Figure S1E) and 2019 seasons (Figure 1E). On the other hand, an opposite effect was noticed for the concentrations of other soil cations, included K^+^ (Figure S1F and Figure 1F), Ca^2+^ (Figure S1G and Figure 1G), and Mg^2+^ (Figure S1H and Figure 1H) in 2018 and 2019, respectively. Although saline water usage significantly decreased soil content of K^+^, Ca^2+^, and Mg^2+^ (*p*_Water_ < 0.0001 for all cations in both seasons), the levels of these cations markedly increased upon the treatment with PGPR and/or biochar regardless of the type of irrigation water.

#### 2.1.2. Activity of Soil Enzymes

The activity of soil urease (mg NH_4_^+^ g^−1^ dry soil d^−1^) and dehydrogenase (mg TPF g^−1^ dry soil d^−1^) enzymes was significantly reduced after using saline water to irrigate maize plants growing in sodic-saline soil during 2018 (Table S1) and 2019 (Table 1). Nevertheless, the application of PGBP and/or biochar considerably alleviated the negative effect of saline water on the activity of soil enzymes. For instance, in freshwater-irrigated soil, the application of PGPR + biochar together significantly increased the activity of urease (220.15 ± 2.41 and 229.32 ± 3.51 mg NH_4_^+^ g^−1^ dry soil d^−1^ in 2018 and 2019, respectively) and dehydrogenase (146.78 ± 4.07 and 156.14 ± 3.34 mg TPF g^−1^ dry soil d^−1^ in 2018 and 2019, respectively) compared with non-treated controls.

Likewise, the activities of urease (158.14 ± 2.37 and 165.51 ± 3.05 mg NH_4_^+^ g^−1^ dry soil d^−1^ in 2018 and 2019, respectively) and dehydrogenase (117.89 ± 1.86 and 121.44 ± 1.00 mg TPF g^−1^ dry soil d^−1^ in 2018 and 2019, respectively) were significantly induced by dual application of PGPR + biochar to saline water-irrigated soils compared with non-treated control (Table S1 and Table 1). The singular application of biochar showed a higher positive impact on the enzymatic activity of both urease and dehydrogenase enzymes compared with singular PGPR application (Table S1 and Table 1).

#### 2.1.3. Bacteriological Characteristics

Soil microbiota structure significantly reduced under saline water irrigation (Table S1 and Table 1) compared to freshwater irrigation during the two growing seasons. There was a significant interaction between the type of irrigation water and soil amendments in the 2018 and 2019 seasons (*p*_Water × Treatment_ < 0.0001 in both seasons) in terms of the total counts of bacteria, *Azotobacter* sp., and *Bacillus* spp. Briefly, the soil microbial population was significantly reduced at 80 days post-seed sowing differed significantly with regard to soil amendments and irrigation water during 2018 and 2019.

The utilization of saline water to irrigate maize plants significantly affected the total counts of bacteria (1.54 ± 0.05 and 2.35 ± 0.04 Log cfu g^−1^), *Azotobacter* sp. (0.62 ± 0.03 and 0.63 ± 0.02 Log cfu g^−1^), and *Bacillus* spp. (1.02 ± 0.01 and 1.07 ± 0.02 Log cfu g^−1^) compared with non-treated freshwater irrigated soil during 2018 (Table S1) and 2019 (Table 1), respectively. However, the dual application of PGPR and biochar to freshwater-irrigated significantly enhanced the total counts of bacteria (5.87 ± 0.10 and 6.02 ± 0.07 Log cfu g^−1^), *Azotobacter* sp. (2.12 ± 0.02 and 2.13 ± 0.01 Log cfu g^−1^), and *Bacillus* spp. (3.71 ± 0.04 and 3.75 ± 0.04 Log cfu g^−1^) during 2018 and 2019, respectively, compared with non-treated soil.

Likewise, in saline water-irrigated soil, the combined application of PGPR and biochar significantly increased the total counts of bacteria (3.27 ± 0.08 and 5.25 ± 0.04 Log cfu g^−1^), *Azotobacter* sp. (1.85 ± 0.03 and 1.89 ± 0.01 Log cfu g^−1^), and *Bacillus* spp. (2.85 ± 0.04 and 2.95 ± 0.03 Log cfu g^−1^) during 2018 and 2019, respectively, compared with non-treated soil. Generally, the combined application of PGPR and biochar significantly increased the total counts of common soil microbial groups followed by singular application of biochar in both seasons regardless of the type of irrigation water (Table S1 and Table 1).

### 2.2. Soil Amendment Using Biochar and PGPR Enhanced Maize Performance and Resilience to Water Salinity

#### 2.2.1. Leaf Area Index and Photosynthetic Pigments

**Figure 2:**
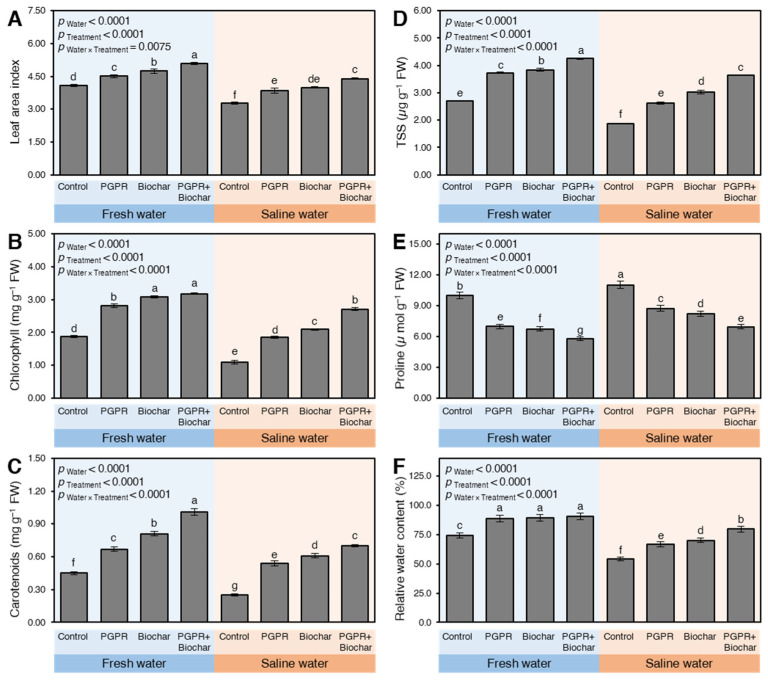
Leaf area index, photosynthetic pigments, and biochemical traits and of maize plants growing in sodic-saline soil and irrigated with fresh and saline water after the application of biochar and PGPR during the 2019 season. Data presented are the means ± standard deviation (mean ± SD) of three biological replicates. Presented pairwise differences connecting letters (significance letters) were generated based on the *p*-value of the interaction between water type (as the main plots) and treatments (as subplots) that were mentioned as (*p*_Water × Treatment_). Means followed by different letters indicate statistically significant differences among treatments according to Tukey’s honestly significant difference (HSD) test (*p* ≤ 0.05), whereas means followed by the same letters indicate no statistically significant differences among them. TSS: Total soluble sugar.

Generally, the Leaf area index (LAI) of maize leaves significantly decreased (*p*_Water_ < 0.0001) when plants were irrigated with saline water during 2018 (Figure S2A) and 2019 (Figure 2A). These subsequent negative effects were significantly diminished upon treating maize plants with PGPR and/or biochar. Plants irrigated with saline water and treated with PGPR + biochar had slightly higher LAI than control plants irrigated with freshwater during 2018 (*p*_Water × Treatment_ = 0.0075; Figure S2A) and 2019 (*p*_Water × Treatment_ = 0.0412; Figure 2A). Interestingly, although the singular application of PGPR and biochar differed significantly during 2018, no significant differences were observed between them in 2019.

Similar to LAI, the endogenous content of photosynthetic pigments (total chlorophyll and carotenoids) significantly reduced under irrigation with saline water compared to irrigation with freshwater in sodic-saline soil during 2018 (Figure S2B and Figure 2B) and 2019 (Figure S2C and Figure 2C). For example, the lowest chlorophyll and carotenoids content were obtained from control plants irrigated with saline water in both growing seasons. However, the negative effects of irrigation with saline water on photosynthetic pigments were significantly lessened when maize plants were treated with the PGPR, biochar, or their combination.

Under both types of irrigation water, PGPR + biochar application increased the total chlorophyll and carotenoids content compared to non-treated control plants (*p*_Water × Treatment_ < 0.0001 for both variables in both seasons). Yet, no significant differences were observed nither in the total chlorophyll content between biochar and PGPR + biochar in 2018 (Figure S2B) nor total carotenoid content between PGPR and biochar treatments in 2019 (Figure 2C).

#### 2.2.2. Total Soluble Sugars (TSS), Proline, and Relative Water Content (RWC)

Even though non-treated control plants severely suffered from saline water irrigation recording the lowest soluble sugars (TSS) content in both seasons (Figure S2D and Figure 2D), the application of PGPR and/or biochar dramatically increased TSS in maize plants irrigated with both types of irrigation water (*p*_Treatment_ < 0.0001 in both seasons). Moreover, saline water-irrigated plants that received PGPR + biochar had significantly higher TSS content than plants irrigated with freshwater without biochar and PGPR application during the 2018 and 2019 seasons (*p*_Water × Treatment_ < 0.0001 in both seasons). The singular application of biochar displayed the second inductive effect on TSS under either fresh or saline water supply followed by PGPR by itself.

On the other hand, the endogenous proline content was significantly increased in non-treated plants as a response to saline water irrigation (*p*_Water_ < 0.0001) during 2018 (Figure S2E) and 2019 (Figure 2E). The results revealed that the plants irrigated with saline water and treated with PGPR + biochar possessed lower proline content than in the case of the individual application of PGPR or biochar during 2018 and 2019. Furthermore, the plants irrigated with freshwater in the presence of PGPR + biochar showed a further reduction in proline content compared with irrigation with saline water during 2018 and 2019 (*p*_Water × Treatment_ < 0.0001 in both seasons).

The RWC of maize leaves significantly declined upon irrigation with saline water compared to freshwater (*p*_Water_ < 0.0001 in both seasons) during the two growing seasons 2018 and 2019 (Figure S2F and Figure 2F, respectively). However, the negative effects of irrigation with saline water were significantly alleviated when maize plants were treated with PGPR, biochar, or their combination (*p*_Treatment_ < 0.0001 in both seasons). It is worth mentioning that, under freshwater irrigation conditions, all treatment (PGPR, biochar, and their combinations) significantly enhanced the RWC without significant differences between them. However, more significant differences were observed between the three treatments and control when maize plants were irrigated with saline water (*p*_Water × Treatment_ < 0.0001 and 0.0002 in 2018 and 2019, respectively).

#### 2.2.3. The Leaf Content of Na^+^, K^+^, and K^+^/Na^+^

**Figure 3:**
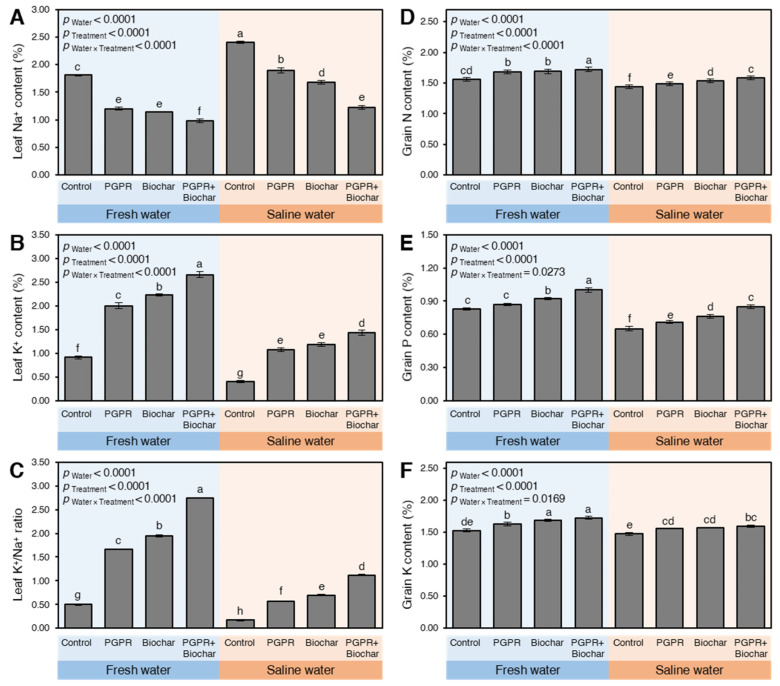
The leaf content of Na^+^ and K^+^, K^+^/Na^+^ ratio, and the NPK content of grains of maize plants growing in sodic-saline soil and irrigated with fresh and saline water after the application of biochar and PGPR during the 2019 season. Data presented are the means ± standard deviation (mean ± SD) of three biological replicates. Presented pairwise differences connecting letters (significance letters) were generated based on the *p*-value of the interaction between water type (as the main plots) and treatments (as subplots) that were mentioned as (*p*_Water × Treatment_). Means followed by different letters indicate statistically significant differences among treatments according to Tukey’s honestly significant difference (HSD) test (*p* ≤ 0.05), whereas means followed by the same letters indicate no statistically significant differences among them.

The leaf Na^+^ content was notably increased when plants were irrigated with saline water in the absence of PGPR and/or biochar (Figure S3A and Figure 3A during 2018 and 2019, respectively). On the contrary, the K^+^ content (Figure S3B and Figure 3B during 2018 and 2019, respectively) and K^+^/Na^+^ ratio (Figure S3C and Figure 3C during 2018 and 2019, respectively) in maize leaves significantly declined upon irrigation with saline water. Interestingly, the application of PGPR and/or biochar significantly influenced the Na^+^, K^+,^ and K^+^/Na^+^ in the leaves (*p*_Treatment_ < 0.0001 for the three variables in both seasons). Soil amendment using biochar and/or PGPR significantly reduced the Na^+^ content and increased K^+^ which resulted in a higher K^+^/Na^+^.

Under both types of irrigation water, the dual application of PGPR + biochar was the best followed by the singular application of biochar and lastly PGPR. For instance, the lowest Na^+^ content (*p*_Water × Treatment_ < 0.0001 and 0.0022 in 2018 and 2019, respectively) and the highest K^+^ content (*p*_Water × Treatment_ < 0.0001 in both seasons) and K^+^/Na^+^ ratio (*p*_Water × Treatment_ < 0.0001 in both seasons) were detected in plants treated with biochar + PGPR upon their irrigation with either fresh or saline water compared to the sole application or control.

#### 2.2.4. The NPK Content of Maize Grains

Irrigation maize plants with saline water in sodic-saline soil significantly declined the N (Figure S3D and Figure 3D), P (Figure S3E and Figure 3E), and K content in maize grains (Figure S3F and Figure 3F) in 2018 and 2019, respectively. However, the negative effects of saline water were significantly alleviated when maize plants were treated with biochar, PGPR, or their combination. PGPR and biochar application significantly induced the NPK in maize grains regardless of the type of irrigation water. Additionally, the highest N, P, and K contents were attained from plants treated with PGPR + biochar when irrigated with either fresh or saline water in sodic-saline soil during 2018 and 2019.

#### 2.2.5. Yield and Yield Components of Maize

In the absence of PGPR and/or biochar treatments, the maize yield and its components, including the number of grains per ear, 100-grain weight (g), grain yield (ton/ha), stover yield (ton/ha), and harvest index (%), was significantly decreased upon irrigation with saline water compared to freshwater during 2018 (Table S2) and 2019 (Table 2) seasons. However, these negative effects of saline water were significantly attenuated when maize plants were treated with biochar, PGPR, or their combination.

During the 2018 season, the highest grains per ear, 100-grain weight, grain yield, and stover yield corresponded to plants treated with the PGPR + biochar and irrigated with freshwater during 2018 (Table S2) and 2019 (Table 2) seasons. Interestingly, during the 2018 season, the highest harvest index was recorded by saline water-irrigated plants when treated with PGPR + biochar (36.22 ± 0.27%) followed by freshwater-irrigated plants when treated with biochar only (36.14 ± 0.47%), with no significant differences between them.

However, during the 2019 season, PGPR + biochar-treated plants had the highest harvest index when irrigated with freshwater (36.66 ± 0.14%) or saline water (35.97 ± 0.63%). In other words, no significant differences in HI between plants irrigated with either fresh or salty water when PGPR + biochar treatment was applied in both seasons. These results revealed that the application of PGPR and/or biochar could substantially mitigate the adverse impacts of irrigating maize plants using saline water in sodic-saline soil.

## 3. Discussion

The increase in the world’s population and high demand for better nutritional and commercial quality foods are driving us towards optimizing the use of our natural resources to ensure food safety and security. In the current study, we aimed to underline the potential application of PGPR and/or biochar as a sustainable eco-friendly strategy to improve the resilience of maize plants grown in a sodic-saline soil and irrigated with saline water. It was reported previously that the reduction in plant growth occurs when salts exist at a sufficiently high content in the root zone; that directly and indirectly, injure plant tissues [35].

The osmotic effect of salinity impairs plant growth due to the shortage in water uptake. Furthermore, upon salinity exposure plants experience ion toxicity of the increased uptake and accumulation of Na^+^ and Cl^-^ in plant tissues [10,36,37]. Moreover, elevated Na^+^ and Cl^-^ concentrations in soil solution antagonistically inhibit the uptake of essential nutrients, such as K^+^, Ca^2+^, Mg^2+^, and NO_3_^-^, disrupting cell ion homeostasis and inhibiting photosynthesis, enzyme activity, and protein synthesis, destroying chloroplasts, and causing nutritional disorders [10,36,37].

In higher plants, salinity stress usually slows the photosynthesis rate due to the lack of CO_2_ availability, degradation of photosynthetic pigments, and reduced leaf area [10,38]. As a response, stressed plants reduce their water loss via decreasing their stomatal conductance, which consequently limits CO_2_ diffusion [39]. Moreover, Marcelis and Van Hooijdonk attributed an 80% reduction in radish growth to the decrease in leaf area that diminishes light interception, while the other 20% was ascribed to a decline in stomatal conductance [40].

Nevertheless, the application of PGPR and/or biochar significantly induced plant performance in sodic-saline soil. Despite the fact that the singular application of PGPR or biochar substantially improved plant growth under both fresh and saline water irrigation, the combined application of PGPR + biochar resulted in the highest improvement in soil properties and, consequently, the growth and productivity of maize. The positive effect of PGPR application on soil physicochemical properties may be attributed to the enhancement in soil structure and soil particulates due to the excreted polysaccharides from microbial cells [41].

Consequently, an improvement in the soil water holding capacity, porosity, aeration, and infiltration occurs. This would facilitate easy penetration of plant roots to deeper soil profiles to reach the subsurface layer that could be less saline. The synergistic effect of PGPR and biochar on soil biota and their activities is due to a further biostimulation effect [41] that increases the activity of soil microbes in the rhizosphere. This eventually results in the better growth of maize. The higher activities of soil enzymes, owing to biochar application as well as PGPR, could be explained by the contribution of biochar to enhance soil nutrient availability, physicochemical traits, and the interaction with extracellular soil enzymes.

The relationship between soil pH and ESP has been proven previously. Briefly, high Na^+^ concentration inhibits the uptake of other elements, such as Ca^2+^, K^+^, and other cations, directly via antagonism or indirectly. The direct inhibition of cations by high Na^+^ levels might be due to the antagonistic relationship between them. On the other hand, high Na^+^ levels indirectly inhibit cations uptake via increasing soil pH that reduces the phytoavailability of most nutrients.

However, the generation of stable soil aggregates by either binding ions, such as Ca^2+^ and Mg^2+^ or the excreted polysaccharides from soil microbes would be the first step in reclaiming this soil and supporting plant growth. In the present study, the application of PGPR + biochar significantly lowered the ESP and Na^+^ and, on the other hand, increased the concentrations of K^+^, Ca^2+^, and Mg^2+^ in soil solution [4,42].

As a result of its richness in many nutrients, i.e., K^+^, Ca^2+^, and Mg^2+^ in addition to N and P, biochar has the potential to alter the composition of soil solution; consequently, it affects the soil structure. The high content of Ca^2+^ and Mg^2+^ in the soil solution allows the substitution of Na^+^ on the surface of soil aggregates leading to a higher rate of Na^+^ leaching and, therefore, soil salinity decreases [43]. Therefore, an improvement in soil physical properties occurred resulting in better development of maize plants in sodic-saline soil irrigated with saline water.

The PGPR + biochar treatment increased the content of total chlorophyll, carotenoids, and TSS and decreased the proline content; moreover, it enhanced the RWC and LAI. The positive effect of PGPR + biochar is attributed to maintaining water uptake and alleviating the imbalanced nutrients. The addition of biochar improves the nutrient balance in soil solution through discharging the mineral nutrients, especially K^+^, Ca^2+^, and Mg^2+^. On the other hand, it decreases Na^+^ uptake, augmenting the K^+^/Na^+^ ratio [44]. Likewise, PGPR possibly diminished the absorption of Na^+^ by plant roots through the excretion of indol-3-acetic acid (IAA) and bacterial exopolysaccharides, which can bind to Na^+^ and lessen its uptake and accumulation in plant tissues [45].

The total chlorophyll, carotenoids, and TSS are considered suitable indicators for plant health under irrigation with saline water and salt-affected soil [46]. The reduction in the content of total chlorophyll, carotenoids, and TSS under salinity stress is mostly triggered by chloroplast impairments, which cause physiological changes leading to a reduction in plant development and productivity [46]. However, the application of PGPR + biochar increased the total chlorophyll, carotenoids, and TSS. This depends on the adequate supply of nutrients under such abiotic stress, especially N.

The proper N content in plant leaves is essential for better plant development; it also expands the leaf area for higher light interception leading to higher rates of photosynthesis [47]. The RWC is a vital tool that is directly connected to soil water status and potential productivity [48]. Salinity stress decreases water productivity by reducing the RWC as a result of soil osmotic pressure that impairs water uptake [10]. In the present study, the reduction in RWC was accompanied by an increase in proline content and a decrease in photosynthesis, nutritional balance, and grain yield [48].

The stimulatory effects of PGPR can be improved more by the addition of biochar [49]. This perhaps is a result of the high water holding capacity of biochar [50]. For instance, Ahmad et al. reported that the combined application of biochar and PGPR enhanced soil moisture content; this could cause a dilution impact on the soil solution decreasing its osmotic pressure [51]. Thus, decreasing osmotic stress helps plants to avoid losing turgor under saline water irrigation in sodic-saline soil resulting in an increase of RWC [52,53]. It has been affirmed that proline content is adversely linked with RWC under saline water irrigation and soil salinity [52].

In the present study, treatment with PGPR + biochar under freshwater irrigation resulted in the highest N, P, and K uptake by maize plants. Regarding the type of irrigation water, saline water was on par with freshwater under sodic-saline soil conditions. Briefly, the performance of saline water-irrigated maize plants that received PGPR + biochar application was comparable to the performance of those non-treated freshwater-irrigated ones in both seasons. Similar results were reported by Hafez et al. [54]. The results obtained from the present experiment showed that the synergistic use of biochar and PGPR increased N, P, and K uptake substantially more than the singular application of any of them. Akhtar et al. [47] reported that biochar and PGPR had a crucial impact on the formation and stabilization of soil aggregates under saline water irrigation and salt-affected soil.

The highest grain yield (5.8 ± 0.01 ton/ha) and stover yield (10.47 ± 0.11 ton/ha), in the present study, corresponded to plants irrigated with freshwater in presence of PGPR + biochar. The productivity of maize depends mainly on yield components like the number of grain ear^−1^ and 100-grain weight. Freshwater promoted LAI and plant growth; conversely, saline water declined yield-related traits, thus, limiting crop productivity [55,56]. The reduced crop yield upon irrigation due to saline water may be ascribed to the decline in LAI, TSS, RWC, and total chlorophyll, which may eventually result in a decrease of the photosynthesis rate and reduced grain yield and harvest index [51,57].

The synergistic application of PGPR and biochar had a highly positive effect on physiological traits, plant growth, and yield-related traits in comparison to sole application and control treatment. The improvement in plant development might be attributed to the biosynthesis of phytohormones, like IAA, which could essentially be linked to the yield and its components [58], increasing nutrients [48,59,60], the activity of 1-aminocyclopropane-1-carboxylate (ACC) deaminase, and osmolyte production [41,61]. Our findings showed that singular application of biochar significantly enhanced the growth, physiology, and productivity of maize compared with PGPR and non-treated control.

In conclusion, PGPR + biochar significantly improved the soil physical, chemical, and biological properties. PGPR can improve soil physical properties, such as the soil structure, porosity, aeration, and infiltrations, as a result of the enhancement of soil aggregates due to the excreted polysaccharides. They enhanced K^+^ uptake while reduced Na^+^ leading to a higher K^+^/Na^+^ ratio. Under salinity stress plants suffer from osmotic pressure and imbalanced nutrients that have adverse impacts on plant performance, such as photosynthesis, protein synthesis, enzyme activity, and water productivity. Thus, the application of biochar, which has a high water-holding capacity, can dilute the soil solution resulting in lower osmotic stress and consequently enhance the nutrient and water uptake.

## 4. Materials and Methods

### 4.1. Source of PGPR and Growth Conditions

In the present study, two presumptive strains of *Azotobacter chroococcum* SARS 10 and *Pseudomonas koreensis* MG209738 were used. They were previously selected based on their potential as PGPR in a laboratory experiment to produce indole-3-acetic acid (IAA) and for phosphate solubilization as well as ameliorating the seed germination and growth of rice (*Oryza sativa*) under elevated salinity stresses [54]. These strains were provided by the Department of Agricultural Microbiology, Soils, Water and Environment Research Institute (SWERI), Agricultural Research Centre (ARC), Egypt. Jensen’s Medium was used for growing *A. chroococcum* [62], and King’s B broth medium was used to grow P*. koreensis* [63]. PGPR strains were prepared by adding 15 mL of 10^8^ CFU mL^−1^ from fresh cultures of *A. chroococcum* SARS 10 and *P. koreensis* MG209738 to 30 g of sterilized peat moss:vermiculite (1:1) carrier and kept in the fridge for further use.

### 4.2. Biochar Characterization

Biochar was prepared from rice husks and corn stalks at ratio 1:1 by slow pyrolysis in absence of oxygen at 350 °C for 3 h (International Biochar Initiative, 2014). The physicochemical properties of produced biochar were previously reported [54]. Before its application, prepared biochar was powdered in a stainless steel grinder and passed through ~2 mm mesh to remove large particles. One week before transplanting and during the tillage process, biochar was broadcasted to each plot and mixed thoroughly with the surface layer of soil (0–20 cm depth) at a rate of 1.0 kg biochar m^−2^, which is equivalent to 10 ton ha^−1^. Neither the control treatment nor the individual PGPR treatment received biochar.

### 4.3. Field Experiments and Growth Conditions

#### 4.3.1. Location and Treatments

Two field experiments were set up at the Sakha Agricultural Research Station (SARS) Farm, Kafr El-Sheikh, Egypt (Latitude: 31°6′ N and Longitude: 30°56′ E) during two consecutive summer growing seasons of 2018 and 2019 to study the impact of inoculation with PGPR (i.e., *A. chroococcum* SARS 10 and *P. koreensis* MG209738) and/or biochar application under two types of irrigation water (i.e., freshwater and saline water) on the growth, physicochemical properties, soil enzymes, physiological traits, and yield of maize (*Zea mays* L., cv. Hybrid 10) in sodic-saline soil.

Each experimental unit (3 × 4 m) consisted of five ridges 4 m in length and 60 cm apart; the grains were planted at a rate of two to three grains per hole with 20 cm spacing in between, and the space between replications was 1 m. Grains of maize were provided by the Maize Research Department, Sakha, Kafr El-Sheikh, Egypt. The seeding rate was 30 kg ha^−1^, and seeds were planted on 1 June in 2018 and 30 May in 2019. Before seed sowing, grains were inoculated by a mixture (1:1) of the two PGPR strains (prepared as described above) at a rate of 950 g ha^−1^. One week after seed germination, thinning was done to retain one seedling per hole.

#### 4.3.2. Soil Sampling

Soil samples collected from 0–30 cm were air-dried, crushed, and passed through a 2 mm sieve for physicochemical properties analysis (Table S3). The characteristics of irrigation water (Table S4) have been provided by Soil Improvement and Conservation Department, Agricultural Research Center, Giza, Egypt.

#### 4.3.3. Agronomic Practices

Maize plants received the following amounts of irrigation water (fresh or saline water) during the following different growth stages: sowing (1350), tillering (2150), elongation (1070), anthesis (1350), and filling (780) in m^3^ per hectare as recommended by Gharib et al. [64]. Same amounts were given during both growing seasons. All other agronomic practices were done as recommended by the Ministry of Agriculture and Land Reclamation, Egypt as follows: phosphorus in the form of calcium superphosphate (15.5% P_2_O_5_), and potassium in the form of potassium sulfate (48% K_2_O) were broadcasted and incorporated during soil tillage at the rates of 360 kg ha^−1^ and 120 kg ha^−1^, respectively. Nitrogen fertilizer was added in the form of ammonium nitrate (33.5% N) at 288 kg ha^−1^ in two equal doses before the first and the second irrigations from seed sowing.

### 4.4. Plant Biometrics

#### 4.4.1. Preparation of Plant Samples

Plant samples were washed thoroughly by 0.1 M HCl, rinsed in deionized water to remove the adhered soil particles and/or other substances, and then left to dry under laboratory conditions. Air-dried plant tissues were placed in a forced-air oven (Binder Model ED115, Tuttlingen, Germany) at 60 °C for two days to obtain the dry mass of plant samples. Afterward, dried samples were powdered using stainless steel mill, passed through a 60 mesh screen, and kept in polyethylene pages for further analysis.

#### 4.4.2. Photosynthetic Pigments

Photosynthetic pigments (chlorophyll and carotenoids) are responsible for capturing the energy of sunlight for photosynthesis, and they are sensitive to different environmental stresses. Eighty days after seed sowing, the total chlorophyll (Chl) and carotenoids were determined in tissues collected from the second fully-expanded leaf from the plant tip.

The content of the photosynthetic pigments was determined according to Mousa et al. [65]. Briefly, 0.1 g of fresh leaf tissue was ground with 5 mL acetone 80% then centrifuged at 13,000 rpm for 10 min. The absorbance of the supernatant was read at 645, 663, and 470 nm using a spectrophotometer (model UV-160 A, Shimadzu, Japan). The content of chlorophyll and carotenoid (mg g^−1^ FW) in the extract was calculated using Equations (1) and (2), respectively, as below:

Total Chl = 20.21 (A645) + 8.02 (A663)(1)

Carotenoids = (1000 (A470) − 2.27 (Chl *a*) − 81.4 (Chl *b*))/227 (2)

#### 4.4.3. Proline Content

The amino acid, proline, is well known to be associated with the plant response to abiotic stress, such as salinity. Thus, the endogenous proline content in the second fully-expanded leaf from the plant tip was determined as described by Bates et al. [66] after 80 days from seed sowing. Briefly, 0.1 g of fresh plant tissues was thoroughly mixed with 4 mL sulfosalicylic acid (3.0%) in a mortar and left overnight at 5 °C. The suspension was centrifuged at 3000 rpm for 5 min at room temperature.

Four milliliters of acidic ninhydrin reagent were mixed with the supernatant. Tubes were mechanically shaken then heated in a boiling water bath for 1 h. Afterward, the tubes were cooled, and the mixture was extracted with 4 mL of toluene in a separating funnel. The absorbance of the toluene layer was recorded at 520 nm by spectrophotometry. The concentration of the unknown sample was calculated with reference to the standard curve. The final value is an average of nine samples per treatment.

#### 4.4.4. Total Soluble Sugar (TSS)

TSS is a key determinant of photosynthesis quality, and it is affected directly by different abiotic stresses, particularly salinity and low quality water. TSS content was measured using anthrone reagent as described by Ibragimova et al. [67]. For this purpose, 0.1 mL of alcoholic leaf extract was added to 3 mL freshly prepared anthrone reagent, mixed well, and then boiled in a water bath for 10 min. The absorbance was measured at 620 nm. A calibration curve prepared from glucose was used to quantify TSS content in 80-day-aged leaf samples. The second fully expanded leaf from the plant tip was collected for this measurement at a rate of nine leaves per treatment.

#### 4.4.5. Relative Water Content (RWC)

RWC is one of the most proper determinant of water status in stressed-plants and it is a good indicator for the physiological consequence of cellular water deficiency. RWC in maize leaves was measured using leaf discs (6 mm diameter) from 80-day-aged plants. The fresh weight of discs was recorded using a digital electrical balance, then dipped in distilled water at 25 °C for 24 h to measure the turgid weight (TW). The dry weight (DW) of the discs was measured after placing them in a forced-air oven at 80 °C for 24 h. The RWC content was calculated according to Equation (3) as suggested by Barrs and Weatherly [68] as follows:

(3) $$RWC=\frac{FW-DW}{TW-DW}\times 100$$

#### 4.4.6. Determination of Na^+^ and K^+^ in Maize Leaves

Eighty days from seed sowing, nine leaves (the second fully-expanded leaf) from each treatment were sampled and dried in an oven at 70 °C for 48 h. Dried 0.5 g of leaves were grounded into a fine powder then placed into Kjeldahl digestion tubes, and 5 mL of sulfuric acid (H_2_SO_4_, 95–97%, 1.84 kg L^−1^, Merck) was added. Then, the tubes were placed on the heater and the temperature was increased gradually by 5 °C min^−^^1^ to reach 270 °C, then digestion continued at this temperature for 2 h.

One mL of perchloric acid (HClO_4_, 80%, 1.67 kg L^−1^, Merck) was added to the samples after cooling for 30 min and then temperature increased again to 150 °C for an additional 1 h until the digestion solution turned clear. Using ultra-pure water, the volume of the sample was brought to 50 mL in a volumetric flask. According to Temmingho and Houba [69], the Na^+^ and K^+^ contents were determined using an Atomic Absorption Spectrophotometer (AAS, (Perkin Elmer 3300, LOD = 100 ppb).

#### 4.4.7. Leaf Area Index

The leaf area index at 80 days after planting was measured as described by the association of official analytical chemists (AOAC) [70] using the second fully-expanded leaf from the shoot tip. It was measured according to Equation (4) as follows:

(4) $$Leaf\;area\;index=\frac{\;\mathrm{Leaf}\;\mathrm{area}/\mathrm{plant}\;}{\mathrm{land}\;\mathrm{area}/\mathrm{plant}}$$

### 4.5. Maize Productivity

#### 4.5.1. Yield and Yield Components

At harvest, the number of grains ear^−1^ was recorded by counting the number of grains in five ears randomly selected in each subplot, and the 100-grain weight was also calculated using the same five ears. The biological air-dried yield (kg ha^−1^) was measured by harvesting the four central rows in each subplot. The ears of two inner ridges were harvested in each subplot and shelled, and then the grains were weighted and adjusted to 15.5% moisture content to estimate the grain yield (kg ha^−1^). The harvest index (%) was calculated as the ratio of grain yield to biological yield and multiplied by hundred.

#### 4.5.2. NPK Content in Maize Grains

Air-dried grain samples were placed into a forced-air oven for 48 h at 70 °C. The dried samples were powdered using a grinder and kept in plastic bags for further analysis. For N, P, and K determination, powdered grain samples were digested with HNO_3_: HClO_4_ solution (2:1). The N content was determined after AOAC [70], while P content was calorimetrically measured according to Sparks et al. [71]. The K content was determined using AAS (LOD = 100 ppb) [71].

### 4.6. Soil Measurements

#### 4.6.1. Soil Dehydrogenase and Urease Activity

Eighty days from seed sowing, soil samples were collected at 0–20 cm depth to measure the activity of dehydrogenase and urease enzymes. The collected soil samples were passed through a 5 mm mesh to remove the plant remains and big particles, such as stones then kept in polyethylene pages at −20 °C for further analysis. Measurement of urease activity was done based on the quantitative determination of ammonia by the spectrophotometric measurement at 660 nm by Kemper’s method [72]. The dehydrogenase activity was measured as described by Mersi, [73] by mixing the soil samples with INT-solution, incubating them for 2 h at 40 °C. The reduced iodonitro-tetrazolium formazan (INTF) was extracted with dimethyl-formamide and ethanol and measured photometrically at 464 nm.

#### 4.6.2. Microbial Estimations

The total count of bacteria was estimated by soil extract agar medium according to Abdel-Malek and Ishac [74], while the total count of Pseudomonas was measured by King’s B agar medium according to King et al. [63]. The most probable number of *A. chroococcum* was estimated using modified Ashb’s media according to the Cochrane method [75] and calculated using tables of Casida et al. [76]. All microbial estimation was counted at 80 days from seed sowing.

#### 4.6.3. Soil Physicochemical Properties

For the analysis of soil chemical properties, the soil was sampled at 0–30 cm depth at maize harvest using an auger. After air-drying, soil samples were passed through a 2 mm sieve. The EC_e_ (dS m^−1^) was assessed in soil paste extract using EC-meter (Genway, UK), whereas pH was determined in a 1:2.5 soil: distilled water suspension using pH-meter (Genway, UK, relative error; ±0.05). The levels of Na^+^, K^+^, Ca^2+^, and Mg^2+^ ions (meq L^−1^) were evaluated in soil paste extract using AAS (LOD = 100 ppb)[71]. The exchangeable sodium percentage (ESP) was calculated according to Equation (5) as suggested by Seilsepour et al. [77]:

ESP = 1.95 + 1.03 × SAR (R^2^ = 0.92) (5)

where SAR (Sodium adsorption ratio) was calculated using Equation (6) as described by Richards [78]:

(6) $$\mathrm{SAR}=\left[{\mathrm{Na}}^{+}\right]/\sqrt{\frac{\left(\left[{\mathrm{Ca}}^{2}{}^{+}\right]+\;\left[{\mathrm{Mg}}^{2}{}^{+}\right]\right)}{2}\;}$$

where Na^+^, Ca^2+,^, and Mg^2+^ were expressed in milliequivalents per liter (mEq L^−1^).

### 4.7. Statistical Analysis

A full factorial split-plot design arranged in randomized complete blocks was used as an experimental layout throughout the study. Our experimental design consists of two factors: (1) two types of irrigation water (freshwater vs. saline water) as the main plot and (2) four treatments (control, PGPR, biochar, and PGPR+biochar) as subplots. All experiments were repeated twice in two different seasons (2018 and 2019) with at least three biological replicates for each treatment. However, all data of the 2018 season are presented as supplementary materials for this study.

The normality and homoscedasticity of the data were tested, and data transformation was done as necessary. The analysis of variance (ANOVA) was used to test the significant differences among irrigation waters (*p*_Water_), treatments (*p*_Treatment_), and their interaction (*p*_Water × Treatment_). Tukey’s honestly significant difference (HSD) test was used for post-hoc analysis (*p* < 0.05). The presented pairwise differences connecting letters (significance letters) were generated based on the *p*-value of the interaction between water type (as the main plots) and treatments (as subplots) that were mentioned as (*p*_Water × Treatment_). ANOVA and Tukey’s test were carried out using JMP Data analysis software Version 15 [79].

## 5. Conclusions

Our results suggest that the dual application of PGPR and biochar can be an effective and useful tool to enable the utilization of low-quality water and soil, especially in arid and semiarid regions as it considerably ameliorates the hazardous impacts of saline water in sodic-saline soil. The application of PGPR + biochar reduced the soil salinity, which led to inducing photosynthetic pigments and, therefore, the photosynthesis process and finally maize productivity.

Nevertheless, the potentiality of PGPR and biochar varies with the source and species of PGPR used and material types, the pyrolysis of biochar as well as the soil system. The mechanism by which PGPR and biochar mitigate the detrimental impacts of salinity on both plant and soil ecosystems is complex. The economic benefit of preparation PGPR and biochar and the application rate should be intensively studied. Thus, long-term experiments are crucially needed to assess the impact of PGPR and biochar on plants grown in sodic-saline soils.

## Figures and Tables

**Table 1:** The activity of soil dehydrogenase and urease enzymes and count of some microbial groups at 80 days after seed sowing of maize plants irrigated with fresh and saline water in sodic-saline soil after the application of biochar and PGPB during the 2019 season ^§^.

Treatment		Urease (mg NH_4_^+^ g^−1^ Dry Soil d^−1^)	Dehydrogenase (mg TPF g^−1^ Dry Soil d^−1^)	Bacteria (Log cfu g^−1^ Soil)	*Azotobacter* (Log cfu g^−1^ Soil)	*Bacillus* spp. (Log cfu g^−1^ Soil)
Fresh water	Control	128.86 ± 3.60 d	64.01 ± 1.52 e	3.44 ± 0.05 e	0.93 ± 0.01 g	1.74 ± 0.02 e
	PGPB ^†^	165.26 ± 2.65 c	95.85 ± 1.60 c	4.23 ± 0.06 d	1.11 ± 0.01 f	2.14 ± 0.10 d
	Biochar ^‡^	187.38 ± 2.16 b	115.97 ± 1.79 b	5.82 ± 0.07 b	1.63 ± 0.01 c	3.13 ± 0.03 b
	PGPR + biochar ^¥^	229.32 ± 3.51 a	156.14 ± 3.34 b	6.02 ± 0.07 a	2.13 ± 0.01 a	3.75 ± 0.04 a
Saline water	Control	99.29 ± 2.10 e	49.92 ± 2.41 f	2.35 ± 0.04 f	0.63 ± 0.02 h	1.07 ± 0.02 f
	PGPB	121.39 ± 3.57 d	80.65 ± 2.65 d	3.45 ± 0.05 e	1.23 ± 0.02 e	1.75 ± 0.02 e
	Biochar	127.66 ± 2.66 d	94.22 ± 1.64 c	3.54 ± 0.05 e	1.39 ± 0.01 d	1.85 ± 0.03 e
	PGPR + biochar	165.51 ± 3.05 c	121.44 ± 1.00 a	5.25 ± 0.04 c	1.89 ± 0.01 b	2.95 ± 0.03 c
**F-test**						
*p*_Water_		<0.0001	<0.0001	<0.0001	<0.0001	<0.0001
*p*_Treatment_		<0.0001	<0.0001	<0.0001	<0.0001	<0.0001
*p*_Water × Treatment_		<0.0001	<0.0001	<0.0001	<0.0001	<0.0001

^§^ Data presented are the means ± standard deviation (mean ± SD) of three biological replicates. Presented pairwise differences connecting letters (significance letters) were generated based on the *p*-value of the interaction between water type (as the main plots) and treatments (as subplots) that were mentioned as (*p*_Water × Treatment_). Means followed by different letters indicate statistically significant differences among treatments according to Tukey’s honestly significant difference (HSD) test (*p* ≤ 0.05), whereas means followed by the same letters indicate no statistically significant differences among them. ^†^ PGPB (*Azotobacter chroococcum* SARS 10 and *Pseudomonas koreensis* MG209738) added at a 1:1 ratio ^‡^ Biochar is added at the rate of 1.0 kg m^−2^ (10 ton ha^−1^). **^¥^** PGPB at a 1:1 ratio + Biochar at the rate of 1.0 kg m^−2^ (10 ton ha^−1^).

**Table 2:** The yield and yield components of maize plants irrigated with fresh and saline water in sodic-saline soil in the presence of biochar and PGPB during the 2019 season ^§^.

Treatment		Number of Grains Ear^−1^	100-Grain Weight (g)	Grain Yield (ton/ha)	Stover Yield (ton/ha)	Harvest Index (%)
Fresh water	Control	425.27 ± 1.6 d	30.45 ± 0.49 c	4.55 ± 0.04 e	8.86 ± 0.06 e	33.95 ± 0.15 d
	PGPB ^†^	436.69 ± 0.9 b	32.30 ± 0.32 b	5.22 ± 0.03 c	9.76 ± 0.07 c	34.87 ± 0.26 cd
	Biochar ^‡^	437.94 ± 0.9 b	33.55 ± 0.47 b	5.43 ± 0.03 b	9.92 ± 0.04 b	35.36 ± 0.66 bc
	PGPR + biochar ^¥^	446.63 ± 0.9 a	35.77 ± 0.96 a	5.95 ± 0.02 a	10.29 ± 0.04 a	36.66 ± 0.14 a
Saline water	Control	408.83 ± 0.62 f	23.38 ± 0.67 e	4.3 ± 0.05 f	8.43 ± 0.07 f	33.79 ± 0.29 d
	PGPB	421.45 ± 1.25 e	27.13 ± 0.78 d	4.63 ± 0.06 e	8.87 ± 0.04 e	34.32 ± 0.37 cd
	Biochar	424.84 ± 0.82 d	28.32 ± 0.67 d	4.76 ± 0.04 d	8.95 ± 0.03 e	34.70 ± 0.21 cd
	PGPR + biochar	429.65 ± 0.71 c	32.72 ± 0.31 b	5.26 ± 0.02 c	9.36 ± 0.08 d	35.97 ± 0.63 ab
**F-test**						
*p*_Water_		<0.0001	<0.0001	<0.0001	<0.0001	<0.0001
*p*_Treatment_		<0.0001	<0.0001	<0.0001	<0.0001	<0.0001
*p*_Water × Treatment_		=0.0196	=0.0004	<0.0001	<0.0001	=0.0310

^§^ Data presented are the means ± standard deviation (mean ± SD) of three biological replicates. Presented pairwise differences connecting letters (significance letters) were generated based on the *p*-value of the interaction between water type (as the main plots) and treatments (as subplots) that were mentioned as (*p*_Water × Treatment_). Means followed by different letters indicate statistically significant differences among treatments according to Tukey’s honestly significant difference (HSD) test (*p* ≤ 0.05), whereas means followed by the same letters indicate no statistically significant differences among them. ^†^ PGPB (*Azotobacter chroococcum* SARS 10 and *Pseudomonas koreensis* MG209738) added at a 1:1 ratio ^‡^ Biochar is added at the rate of 1.0 kg m^−2^ (10 ton ha^−1^). **^¥^** PGPB at a 1:1 ratio + Biochar at the rate of 1.0 kg m^−2^ (10 ton ha^−1^).

## Data Availability

The data that supports the findings of this study are contained within the article or supplementary material and available from the corresponding author upon reasonable request.

## Supplementary Materials

The following are available online at https://www.mdpi.com/article/10.3390/plants10091960/s1, Table S1: Activity of soil dehydrogenase and urease enzymes and count of some microbial groups at 80 days after seed sowing of maize plants irrigated with fresh and saline water in sodic-saline soil after the application of biochar and PGPR during the 2018 season.; Table S2: Yield and yield components of maize plants irrigated with fresh and saline water in sodic-saline soil in presence of biochar and PGPR during the 2018 season.; Table S3: Physicochemical characteristics of the experimental soil in the two growing seasons 2018 and 2019.; Table S4: Characterization of irrigation water during the 2018 and 2019 growing seasons.; Figure S1: Soil chemical properties at the harvest time of maize plants growing in sodic-saline soil and irrigated with fresh and saline water after the application of biochar and PGPR during 2018 season.; Figure S2: Leaf area index, photosynthetic pigments, and biochemical traits and of maize plants growing in sodic-saline soil and irrigated with fresh and saline water after the application of biochar and PGPR during the 2018 season.; Figure S3: The leaf content of Na^+^, K^+^, and K^+^/Na^+^ ratio, and the NPK content of grains of maize plants growing in sodic-saline soil and irrigated with fresh and saline water after the application of biochar and PGPR during the 2018 season.
