# Supplementary material for: The Integrated Amendment of Sodic-Saline Soils Using Biochar and Plant Growth-Promoting Rhizobacteria Enhances Maize (Zea mays L.) Resilience to Water Salinity
Source: Plants (Basel). 2021 Sep 20;10(9):1960. doi: 10.3390/plants10091960 (PMC8466265; doi:10.3390/plants10091960)
Supplement: Supplementary file 1 [file plants-10-01960-s001.zip › plants-1372310-supplementary.pdf]

plants

Supplementary Materials

The integrated amendment of sodic-saline soils using biochar and plant growth-promoting rhizobacteria enhances maize (*Zea mays* L.) resilience to water salinity

Yasser Nehela [1,2,*], Yasser S. A. Mazrou [3,4], Tarek Alshaal [5,6], Asmaa M. S. Rady [7], Ahmed M. El-Sherif [8], Alaa El-Dein Omara [9], Ahmed M. Abd El-Monem [10], Emad M. Hafez [11]

[1] Department of Agricultural Botany, Faculty of Agriculture, Tanta University, Tanta 31527, Egypt; yasser.nehela@ufl.edu
[2] Citrus Research and Education Center, Department of Plant Pathology, University of Florida, 700 Experiment Station Rd., Lake Alfred, FL 33850, USA.[3] Business Administration Department, Community college, King Khalid University, Guraiger, Abha 62529, Saudi Arabia; ymazrou@kku.edu.sa
[4] Department of Agriculture Economic, Faculty of Agriculture, Tanta University, Tanta 31527, Egypt; yasser.mazroua@agr.tanta.edu.eg
[5] Agricultural Botany, Plant Physiology and Biotechnology Department, University of Debrecen, AGTC. 4032 Debrecen, Hungary; alshaaltarek@gmail.com
[6] Soil and Water Department, Faculty of Agriculture, University of Kafrelsheikh, 33516 Kafr El-Sheikh, Egypt; tarek.ibrahim@agr.kfs.edu.eg [7] Crop Science Department, Faculty of Agriculture (EL-Shatby), Alexandria University, Alexandria 21545, Egypt; asmaa.mohamed@alexu.edu.eg
[8] Department of Agronomy, Faculty of Agriculture, Fayoum University 63514, Egypt; ama16@fayoum.edu.eg
[9] Department of Microbiology, Soils, Water and Environment Research Institute, Agricultural Research Center, Giza 12112, Egypt; ala.emara@yahoo.com
[10] Department of Agronomy, Faculty of Agriculture, New Valley University, New Valley, Elkharrga 72511 Egypt; abdelmonem7@gmail.com
[11] Department of Agronomy, Faculty of Agriculture, Kafrelsheikh University, 33516, Kafr El-Sheikh; Egypt; emadhafez2012@agr.kfs.edu.eg, emadhafez2014@gmail.com.

* Correspondence: yasser.nehela@ufl.edu

plants

MDPI

Table S1: Activity of soil dehydrogenase and urease enzymes and count of some microbial groups at 80 days after seed sowing of maize plants irrigated with fresh and saline water in sodic-saline soil after the application of biochar and PGPR during 2018 season [§].

Treatment		**Urease** (mg TPF g^{-1} dry soil d^{-1})	**Dehydrogenase** (mg NH_4^+ g^{-1} dry soil d^{-1})	**Bacteria** (Log cfu g^{-1} soil)	***Azotobacter*** (Log cfu g^{-1} soil)	***Bacillus* spp** (Log cfu g^{-1} soil)
Fresh water	Control	120.70 ± 2.39 de	60.42 ± 3.06 g	2.79 ± 0.05 c	0.91 ± 0.02 f	1.72 ± 0.02 d
	PGPR[†]	154.44 ± 3.74 c	91.42 ± 1.15 d	3.36 ± 0.04 b	1.74 ± 0.02 c	3.22 ± 0.09 b
	Biochar[‡]	172.44 ± 1.84 b	111.22 ± 2.11 c	3.38 ± 0.03 b	1.76 ± 0.01 c	3.32 ± 0.03 b
	Combined[¥]	220.15 ± 2.41 a	146.78 ± 4.07 a	5.87 ± 0.10 a	2.12 ± 0.02 a	3.71 ± 0.04 a
Saline water	Control	90.33 ± 1.74 f	38.12 ± 2.04 h	1.54 ± 0.05 e	0.62 ± 0.03 g	1.02 ± 0.01 e
	PGPR	113.74 ± 2.18 e	70.26 ± 0.77 f	2.53 ± 0.04 d	1.22 ± 0.02 e	1.66 ± 0.02 d
	Biochar	123.62 ± 3.42 d	84.38 ± 0.74 e	2.74 ± 0.12 c	1.36 ± 0.01 d	1.74 ± 0.04 d
	Combined	158.14 ± 2.37 c	117.89 ± 1.86 b	3.27 ± 0.08 b	1.85 ± 0.03 b	2.85 ± 0.04 c
**F-test**						
	P_{Water}	< 0.0001	< 0.0001	< 0.0001	< 0.0001	< 0.0001
	$P_{\text{Treatment}}$	< 0.0001	< 0.0001	< 0.0001	< 0.0001	< 0.0001
	$P_{\text{Water × Treatment}}$	< 0.0001	= 0.0265	< 0.0001	< 0.0001	< 0.0001

[§] Data presented are means ± standard deviation (mean ± SD) of three biological replicates. Presented pairwise differences connecting letters (significance letters) were generated based on the *p*-value of the interaction between water type (as the main plots) and treatments (as subplots) that were mentioned as ($p_{\text{Water × Treatment}}$). Means followed by different letters indicate statistically significant differences among treatments according to Tukey's honestly significant difference (HSD) test ($p \leq 0.05$), whereas means followed by the same letters indicate no statistically significant differences among them.

[†] PGPR (*Azotobacter chroococcum* SARS 10 and *Pseudomonas koreensis* MG209738) added at a 1:1 ratio

[‡] Biochar is added at the rate of 1.0 kg m^{-2} (10 ton ha^{-1})

[¥] PGPR at a 1:1 ratio + Biochar at the rate of 1.0 kg m^{-2} (10 ton ha^{-1})

Means followed by different letters indicate statistically significant differences among treatments according to Tukey's honestly significant difference (HSD) test ($P \leq 0.05$), whereas means followed by the same letters indicate no statistically significant differences among them.

Table S2: Yield and yield components of maize plants irrigated with fresh and saline water in sodic-saline soil in presence of biochar and PGPR during 2018 season §.

Treatment		Number of Grains ear^{-1}	100-Grain weight (g)	Grain yield (ton/ha)	Stover yield (ton/ha)	Harvest index (%)
Fresh water	Control	416.38 ± 1.10 e	29.16 ± 0.82 cd	4.49 ± 0.03 e	8.66 ± 0.12 d	34.12 ± 0.23 cd
	PGPR†	432.13 ± 1.41 c	31.83 ± 0.52 b	5.16 ± 0.04 c	9.66 ± 0.09 b	34.82 ± 0.52 bc
	Biochar‡	436.76 ± 1.10 b	33.11 ± 0.65 b	5.51 ± 0.02 b	9.74 ± 0.07 b	36.14 ± 0.47 a
	Combined¥	443.04 ± 0.60 a	35.50 ± 0.79 a	5.83 ± 0.01 a	10.47 ± 0.11 a	35.77 ± 0.36 ab
Saline water	Control	403.74 ± 1.48 f	23.39 ± 0.76 e	4.15 ± 0.04 f	8.27 ± 0.11 e	33.42 ± 0.72 d
	PGPR	416.79 ± 1.18 e	27.35 ± 0.56 d	4.61 ± 0.05 d	8.58 ± 0.09 de	34.94 ± 0.25 bc
	Biochar	419.15 ± 0.65 e	28.48 ± 0.93 cd	4.65 ± 0.04 d	8.70 ± 0.11 d	34.85 ± 0.14 bc
	Combined	423.21 ± 0.86 d	29.86 ± 0.22 c	5.23 ± 0.02 c	9.21 ± 0.15 c	36.22 ± 0.27 a
**F-test**						
	P_{Water}	< 0.0001	< 0.0001	< 0.0001	< 0.0001	= 0.0488
	$P_{\text{Treatment}}$	< 0.0001	< 0.0001	< 0.0001	< 0.0001	< 0.0001
	$P_{\text{Water × Treatment}}$	= 0.0002	= 0.0275	< 0.0001	< 0.0001	= 0.0078

§ Data presented are means ± standard deviation (mean ± SD) of three biological replicates. Presented pairwise differences connecting letters (significance letters) were generated based on the *p*-value of the interaction between water type (as the main plots) and treatments (as subplots) that were mentioned as ($p_{\text{Water × Treatment}}$). Means followed by different letters indicate statistically significant differences among treatments according to Tukey's honestly significant difference (HSD) test ($p \leq 0.05$), whereas means followed by the same letters indicate no statistically significant differences among them.

† PGPR (*Azotobacter chroococcum* SARS 10 and *Pseudomonas koreensis* MG209738) added at a 1:1 ratio

‡ Biochar is added at the rate of 1.0 kg m^{-2} (10 ton ha^{-1})

¥ PGPR at a 1:1 ratio + Biochar at the rate of 1.0 kg m^{-2} (10 ton ha^{-1})

Means followed by different letters indicate statistically significant differences among treatments according to Tukey's honestly significant difference (HSD) test ($P \leq 0.05$), whereas means followed by the same letters indicate no statistically significant differences among them.

plants

MDPI

Table S3: Physicochemical characteristics of the experimental soil in the two growing seasons 2018 and 2019

Character		2018	2019
pH (1:2.5 soil:water suspension)			
	0-20	8.22 ± 0.02†	8.28 ± 0.03
Soil depth (cm)	20-40	8.19 ± 0.02	8.21 ± 0.03
	40-60	8.16 ± 0.04	8.18 ± 0.02
Electrical conductivity (ECe, dS m^{-1})¥			
	0-20	5.61 ± 0.01	5.66 ± 0.02
Soil depth (cm)	20-40	5.56 ± 0.02	5.59 ± 0.05
	40-60	5.36 ± 0.03	5.54 ± 0.04
ESP# (%)			
	0-20	22.61 ± 0.42	21.50 ± 0.32
Soil depth (cm)	20-40	22.52 ± 0.02	21.36 ± 0.22
	40-60	22.46 ± 0.04	21.21 ± 0.35
Soil organic matter (g kg^{-1})		11.2 ± 0.03	11.7 ± 0.05
Particle size distribution (%)			
Sand		27.22 ± 1.88	27.17 ± 1.98
Silt		25.23 ± 2.02	25.55 ± 1.99
Clay		47.55 ± 2.32	47.28 ± 2.03
Texture grade		clayey	clayey
Soluble cations (mEq L^{-1}) ¥			
Ca^{++}		7.29 ± 0.87	9.42 ± 0.96
Mg^{++}		5.23 ± 1.32	6.34 ± 1.78
Na^{+}		22.63 ± 3.08	21.08 ± 3.45
K^{+}		0.39 ± 0.02	0.39 ± 0.02
Soluble anions (meq L^{-1}) ¥			
CO_3^{--}		nd‡	nd
HCO_3^{-}		3.34 ± 0.68	3.05 ± 0.56
Cl^{-}		18.21 ± 1.15	16.89 ± 1.23
SO_4^{--}		11.15 ± 3.04	10.74 ± 3.45
Available macronutrients (mg kg^{-1})			
N		9.70 ± 0.91	10.33 ± 1.71
P		8.24 ± 1.33	8.94 ± 1.54
K		344 ± 26.42	387 ± 24.33
Total counts of soil microbes			
Bacteria (CFU ×10^7 g^{-1} dry soil)		33 ± 1.2	39 ± 1.5
Fungi (CFU ×10^4 g^{-1} dry soil)		12 ± 0.9	18 ± 1.1
Actinomycetes (CFU ×10^5 g^{-1} dry soil)		23 ± 1.1	24 ± 1.4

†Standard deviation; ‡ not detected; ¥ measured in soil paste extract; # exchangeable sodium percentage, mEq L^{-1}: milliequivalents per liter.

plants

Table S4: Characterization of irrigation water during 2018 and 2019 growing seasons

Character	2018		2019	
	**Fresh water**	**Saline water***	**Fresh water**	**Saline water***
pH	7.28 ± 0.81	8.36 ± 0.17	7.34 ± 0.76	8.39 ± 0.15
EC (dS m^{-1})	0.55 ± 0.02	3.93 ± 0.09	0.51 ± 0.01	3.97 ± 0.11
SAR	1.46 ± 0.04	7.79 ± 0.23	1.42 ± 0.06	7.88 ± 0.26
Na^+ (*mq* L^{-1})	1.98 ± 0.06	16.3 ± 1.42	1.92 ± 0.04	16.81 ± 1.31
Cl^- (*mq* L^{-1})	3.45 ± 0.07	11.62 ± 0.82	3.38 ± 0.05	11.90 ± 0.91
SO_4^{--} (*mq* L^{-1})	0.12 ± 0.01	7.93 ± 0.21	0.14 ± 0.02	8.23 ± 0.25
NH_4^+ (*mq* L^{-1})	1.71 ± 0.03	2.12 ± 0.06	1.83 ± 0.05	2.33 ± 0.05
COD (*mq* L^{-1})	12.00 ± 0.92	nd¥	11.00 ± 1.09	nd
BOD (*mq* L^{-1})	5.42 ± 0.39	nd	5.22 ± 0.62	nd
SS (*mq* L^{-1})	187 ± 12.5	18 ± 1.5	181 ± 13.1	17 ± 1.6
DS (*mq* L^{-1})	388 ± 32	2940 ± 126	393 ± 35	2922 ± 121

COD: Chemical Oxygen Demand; BOD: Biological Oxygen Demand; SS: Suspended solids; DS: Dissolved solids. *Well water at a depth of 20 m; ¥ not detected.

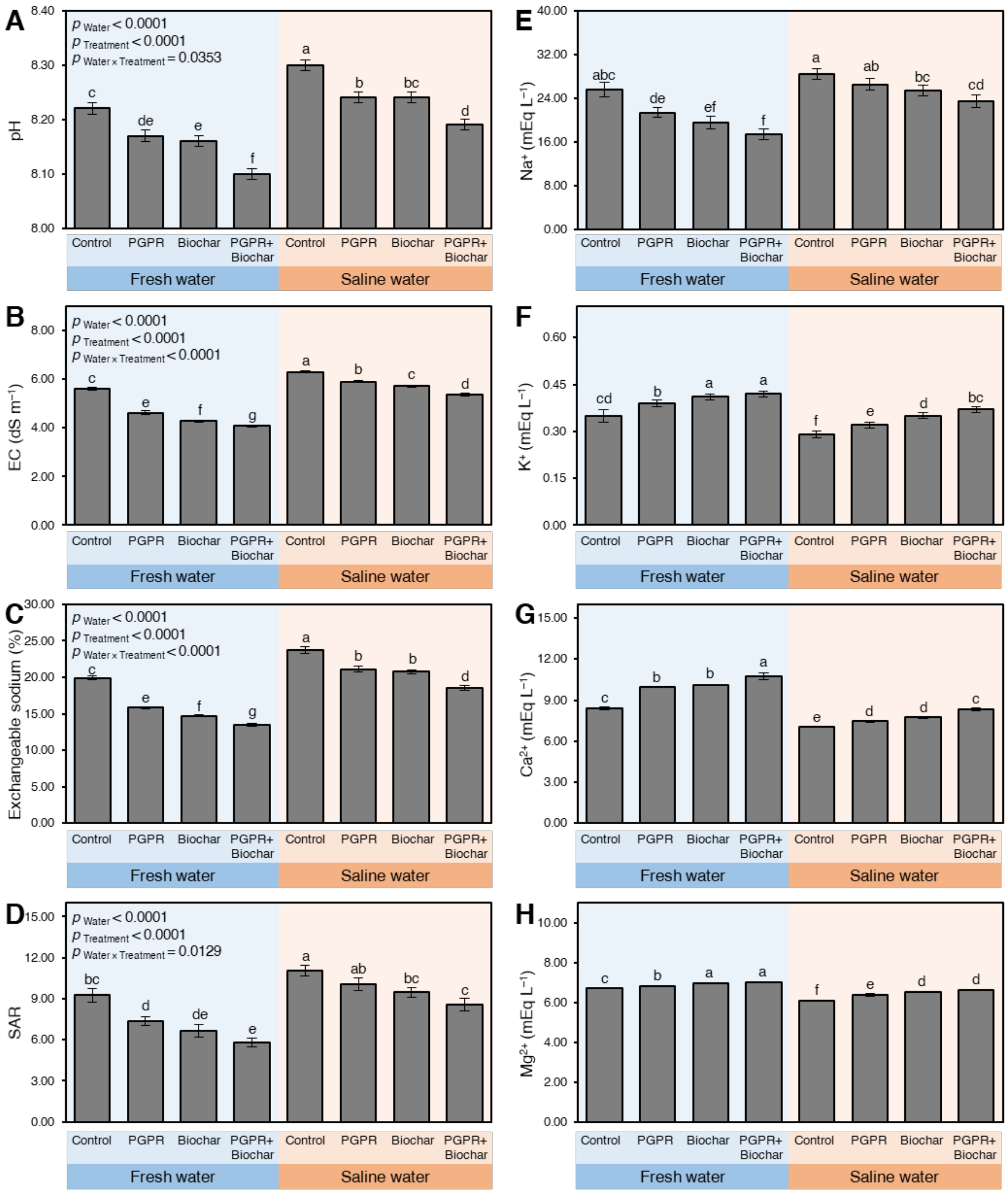

Figure S1: **Soil chemical properties at the harvest time of maize plants growing in sodic-saline soil and irrigated with fresh and saline water after the application of biochar and PGPR during 2018 season**. Data presented are means ± standard deviation (mean ± SD) of three biological replicates. Presented pairwise differences connecting letters (significance letters) were generated based on the *p*-value of the interaction between water type (as the main plots) and treatments (as subplots) that were mentioned as ($p_{Water \times Treatment}$). Means followed by different letters indicate statistically significant differences among treatments according to Tukey's honestly significant difference (HSD) test ($P \leq 0.05$), whereas means followed by the same letters indicate no statistically significant differences among them. EC: Electrical conductivity; SAR: Sodium adsorption ratio; mEq L^{-1}: milliequivalents per liter.

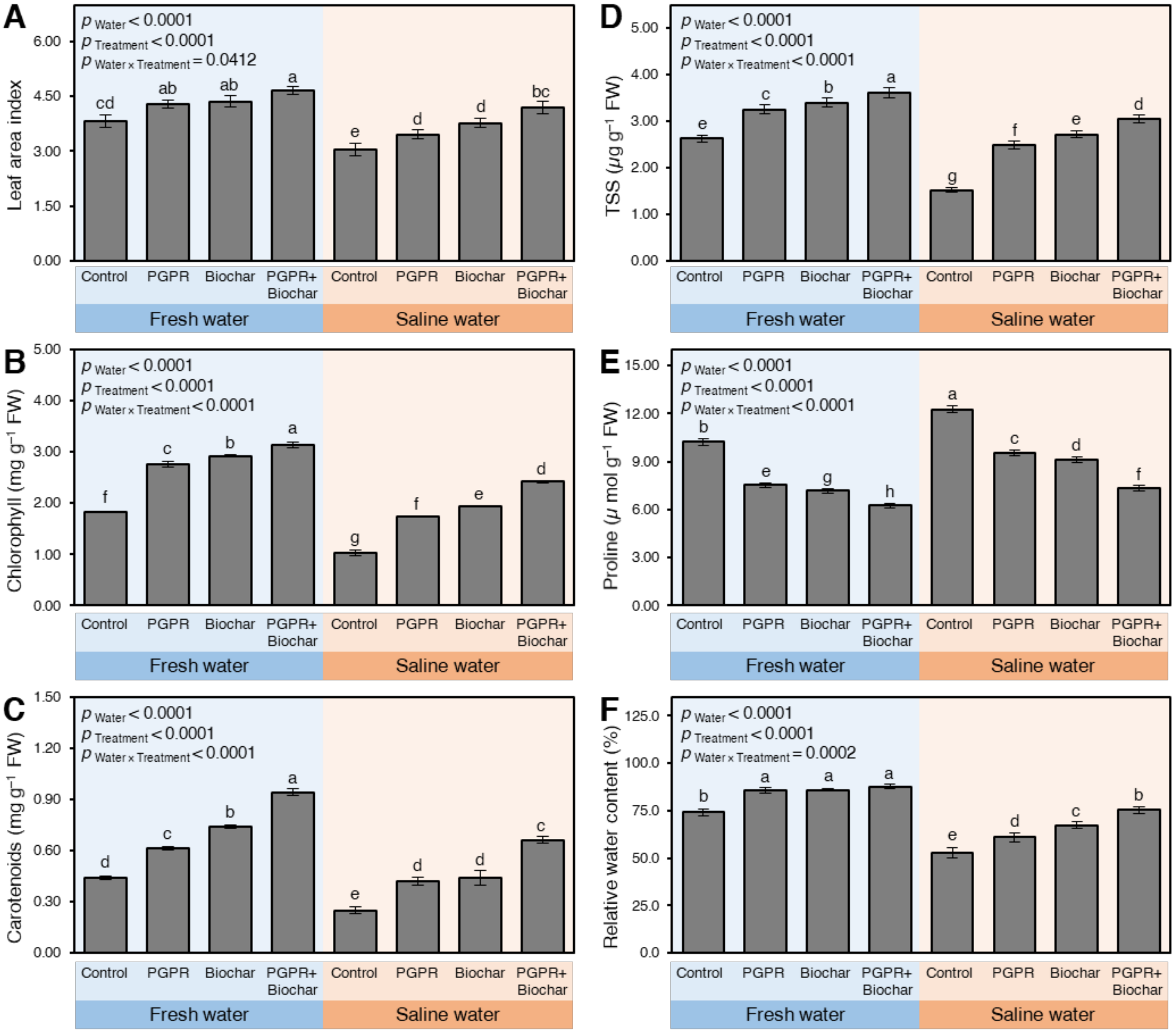

Figure S2: Leaf area index, photosynthetic pigments, and biochemical traits and of of maize plants growing in sodic-saline soil and irrigated with fresh and saline water after the application of biochar and PGPR during 2018 season. Data presented are means ± standard deviation (mean ± SD) of three biological replicates. Presented pairwise differences connecting letters (significance letters) were generated based on the *p*-value of the interaction between water type (as the main plots) and treatments (as subplots) that were mentioned as ($p_{Water \times Treatment}$). Means followed by different letters indicate statistically significant differences among treatments according to Tukey's honestly significant difference (HSD) test ($P \leq 0.05$), whereas means followed by the same letters indicate no statistically significant differences among them. TSS: Total soluble sugar.

plants

MDPI

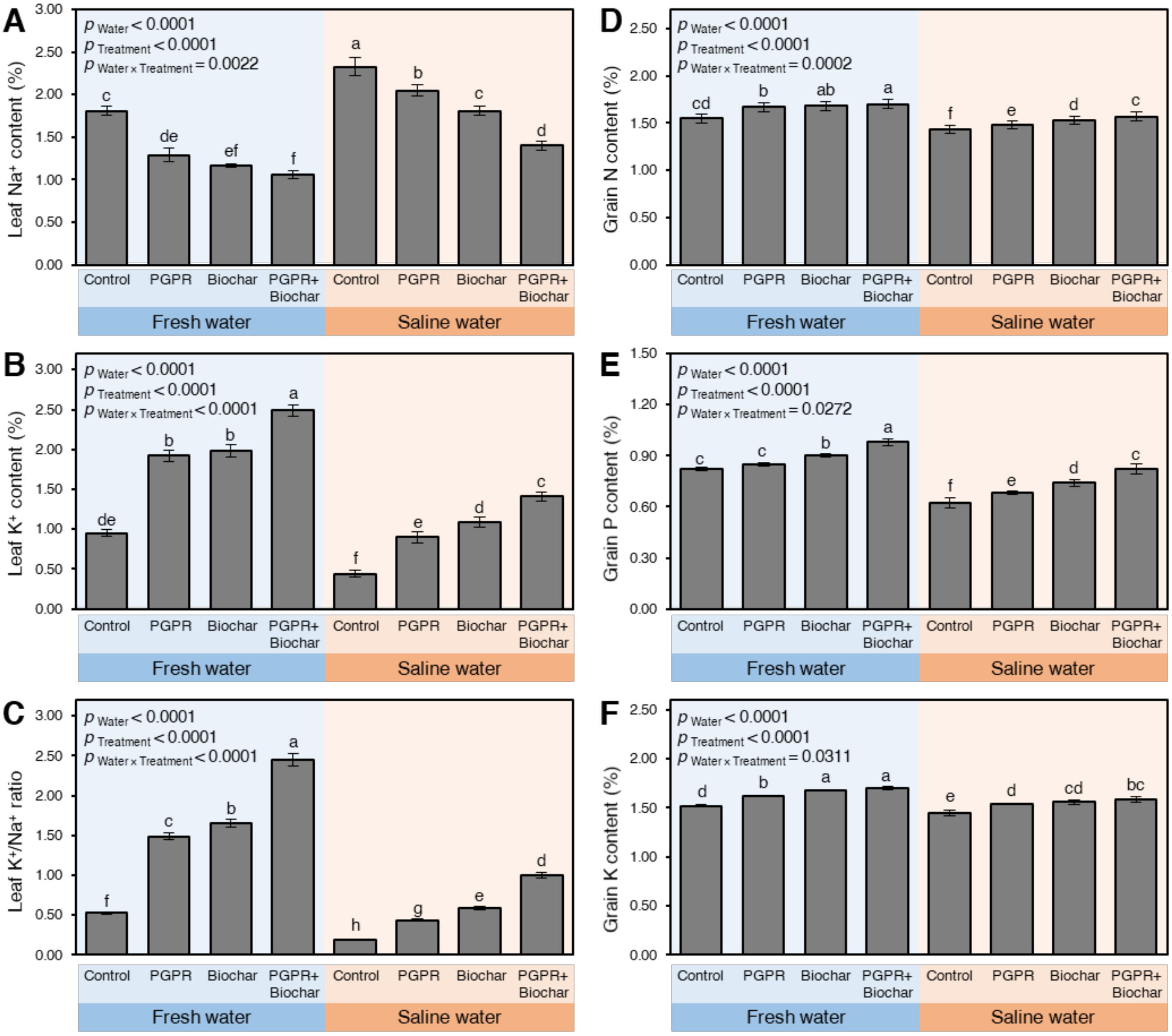

Figure S3: The leaf content of Na^+, K^+, K^+/Na^+ ratio, and the NPK content of grains of maize plants growing in sodic-saline soil and irrigated with fresh and saline water after the application of biochar and PGPR during 2018 season. Data presented are means ± standard deviation (mean ± SD) of three biological replicates. Presented pairwise differences connecting letters (significance letters) were generated based on the *p*-value of the interaction between water type (as the main plots) and treatments (as subplots) that were mentioned as ($p_{Water \times Treatment}$). Means followed by different letters indicate statistically significant differences among treatments according to Tukey's honestly significant difference (HSD) test ($p \leq 0.05$), whereas means followed by the same letters indicate no statistically significant differences among them.
